# Autologous stem cell transplantation for multiple myeloma patients with chronic kidney disease: a safe and effective option

**DOI:** 10.1038/s41409-022-01657-y

**Published:** 2022-04-12

**Authors:** I. Lazana, L. Floro, T. Christmas, S. Shah, K. Bramham, K. Cuthill, P. Bassett, S. Schey, M. Kazmi, V. Potter, A. Pagliuca, M. Streetly, R. Benjamin

**Affiliations:** 1 Department of Haematological Medicine, King’s College Hospital, London, UK; 2 Renal Unit, King’s College Hospital, London, UK; 3 Statsconsultancy Ltd, London, UK; 4 Present Address: Department of Haematology, Guy’s and St Thomas’ Hospital, London, UK

**Keywords:** Myeloma, Medical research

## Abstract

Chronic Kidney Disease (CKD) is a frequent complication in patients with multiple myeloma (MM) and is associated with adverse outcomes. The use of autologous stem cell transplantation (ASCT) has improved disease outcomes, however, the safety and efficacy of ASCT in patients with CKD has been the subject of debate. To investigate this, we conducted a retrospective analysis of 370 MM patients who underwent their first ASCT, including those with mild, moderate and severe CKD as well as normal renal function at the time of transplant. No significant difference in ASCT-related mortality, Progression-Free or Overall Survival was noted between the different renal function groups. A decline in estimated glomerular filtration rate (eGFR) at 1-year of >8.79% was associated with poorer overall survival (*p* < 0.001). The results of this study show that ASCT is a safe and effective option for myeloma patients with CKD, including those on dialysis. Patients who demonstrate renal deterioration at 1-year post-transplant should be closely monitored as this is a predictor for poor survival.

## Introduction

Chronic kidney disease (CKD) and acute kidney injury (AKI) are major complications of multiple myeloma (MM), affecting a third of patients at presentation and nearly 50% at some point during the course of the disease [1, 2]. Approximately 10% of MM patients have advanced CKD requiring long-term dialysis [3]. Renal impairment (RI) is associated with high mortality rates, reaching up to 30% in the first two months post diagnosis of MM [4, 5], as well as increased morbidity resulting in increased healthcare costs and inferior outcomes [1, 6].

Early initiation of anti-myeloma treatment has been shown to have a positive impact on renal function, with a number of studies suggesting that 70% of patients may achieve normalisation of their renal function, resulting in prolonged survival rates [1, 7]. The introduction of novel agents, such as bortezomib, lenalidomide and thalidomide, has been shown not only to be safe in CKD, but also to be associated with a more rapid improvement in renal function [8–11] and improved progression-free survival (PFS) and overall survival (OS) rates [12–14].

ASCT is a well-established consolidation strategy for patients with MM who have achieved remission with induction or salvage treatment. However, its use in patients with CKD has been controversial, due to the perceived risk of renal deterioration secondary to transplant-related toxicity in patients with pre-existing renal impairment. A recent study from Andronesi et al. [6] identified CKD as an independent risk factor for AKI after ASCT for MM, with a significantly higher mortality rate in this subgroup of patients. This, in addition to high dose melphalan-associated toxicities, resulting in higher morbidity and transplant-related mortality (TRM), has led some physicians to preclude patients with advanced CKD from transplant consideration [15–17].

On the other hand, several studies have suggested that ASCT significantly improves the life expectancy and disease outcomes in MM patients with RI [15, 18–20]. Further supporting evidence was provided by a study from Mahindra et al. [21], which showed that ASCT is safe in patients with moderate and severe CKD. Of note, improved outcomes were seen in patients with moderate CKD receiving high dose melphalan at 200 mg/m^2^.

In order to investigate the safety and efficacy of ASCT in MM patients with CKD in the era of novel agents and assess its impact on renal function, we performed a retrospective observational study in patients with MM undergoing ASCT in a large UK transplant centre. The primary objectives of the study were to assess the TRM, PFS and OS in patients with and without CKD and the secondary objective was to evaluate the effect of ASCT on renal function.

## Patients and methods

### Patients

All patients with symptomatic MM, who underwent their first ASCT between Jan 2007 and July 2014 at King’s College Hospital or Guy’s and St Thomas’ Hospital, London, regardless of the number of prior lines of treatment, were included in this study. Patients had achieved at least a minimal response and were transplanted regardless of their renal function. Data collected as part of standard of care evaluation of patients undergoing ASCT were used for this analysis with a data cutoff date of 31/08/2016.

Patients were admitted on Day-2 and received high dose melphalan at 200 mg/m^2^ on Day-1 as part of the conditioning regimen for those with normal renal function, whilst those with an eGFR <50 ml/min/1.73 m^2^ or with additional co-morbidities, frailty and age >70 years received a lower dose of melphalan of 100 or 140 mg/m^2^, at clinician’s discretion. Peripheral blood stem cells were infused 24 or 48 h later (Day 0), in patients with or without CKD, respectively, with a minimum CD34 cell dose of 2 × 10^6^/kg. For dialysis patients, a different transplant protocol was used (Supp Table 2).

Granulocyte colony-stimulating factor was routinely given daily from Day + 7 until stable neutrophil engraftment. Time to neutrophil engraftment was defined as the first of three consecutive days with a neutrophil count >0.5 × 10^9^/L and time to platelet engraftment the first of three consecutive days with an unsupported platelet count >20 × 10^9^/L. Antimicrobial prophylaxis consisted of fluconazole during the period of neutropenia and acyclovir for at least 6 months, with dose adjustment according to renal impairment.

### Evaluation of renal function

Renal function was evaluated based on the eGFR at the time of transplant. eGFR was calculated using the four-variable Modification of Diet in Renal Disease formula [22, 23]. Patients were categorised into four subgroups, based on eGFR in ml/min per 1.73 m^2^: (i) <30, (ii) 30–59, (iii) 60–89 and (iv) ≥90. Based on the criteria suggested by Ludwig et al. [24], renal response was defined as follows: (a) Complete response (CRenal): baseline eGFR ≤50 ml/min/1.73 m^2^ and improvement to ≥60 ml/min/1.73 m^2^ (b) Partial response (PRenal): baseline eGFR <15 ml/min/1.73 m^2^ and improvement to 30–59 ml/min/1.73 m^2^ (c) Minimal response (MRenal): baseline eGFR <15 ml/min/1.73 m^2^ and improvement to 15–29 ml/min/1.73 m^2^, or baseline 15–29 ml/min/1.73 m^2^ and improvement to 30–59 ml/min/1.73 m^2^. Renal function was assessed at D0, D + 100 and at D + 365 post-transplant.

### Myeloma response criteria

Disease response to treatment was defined according to the International Myeloma Working Group (IMWG) response criteria [25] as progressive disease, stable disease, minimal response (MR), partial response (PR), very good partial response (VGPR) or complete response (CR). OS was considered to be the time from stem cell transplant (D0) to death from any cause, and PFS was calculated from D0 until disease progression. PFS2 was defined as the time from first relapse following transplant to occurrence of second relapse. TRM was defined as all deaths that occurred in the first 100 days post ASCT without disease progression.

### Statistical methods

All analyses followed EBMT statistical guidelines [26]. The Kruskal-Wallis test was used for the descriptive analysis. The change in eGFR between D0 and D + 365 was assessed by paired analysis for each patient (individual eGFR change) and compared using the Wilcoxon signed-rank test. The percentage eGFR change was divided into four quartiles to create four equal size patient groups; *Q*_1_: > 15.4% improvement, *Q*_2_: 3.91–15.4% improvement, *Q*_3_: ≤ 8.79% reduction-3.91% improvement and *Q*_4_: > 8.79% reduction. These quartiles were used to analyse the dynamic effect of renal function on survival (OS, PFS and PFS2). Survival curves were calculated by the Kaplan Meier method. Differences in survival were compared by the log-rank test. To assess the effect of eGFR change at 1 year post transplant on survival the time at risk was started at D + 365. TRM was analysed with the competing risk model. Statistical analysis was performed with IBM SPSS version 26 and R version 3.3.1.

## Results

### Patient characteristics

A total of 370 patients with MM were included in the analysis. Data were missing in 36 patients at D + 100 and in four patients at D + 365. 1.6% of patients (*n* = 6) had died by D + 100 and 5.2% (*n* = 17) by D + 365 (Supp Fig. 1). Clinical characteristics of the patients are shown in Table 1. The median age was 60 years (range 32–74), with 22% of patients (*n* = 80) being >65 years. The median time from diagnosis to ASCT was 10.4 months (range 4.6–143.8). 81% of patients (*n* = 296) received one line of induction chemotherapy and 19% (*n* = 70) more than one line prior to transplant. Novel agents were used in the induction regimen in 93% of patients (*n* = 342) with 86% having received thalidomide and/or bortezomib and 7% having received lenalidomide. At the time of transplant, 26% of patients (*n* = 98) were in biochemical CR, 24% (*n* = 88) in VGPR, 31% (*n* = 113) in PR and 5% (*n* = 18) in MR with response status unknown in 14% (*n* = 53) (Table 1).

**Table 1:** The clinical characteristics of 370 MM patients who underwent ASCT between 2007 and 2014.

Patients	Total (*n* = 370)	eGFR (ml/min/1.73 m^2^)				
		<30		30–59 (*n* = 42)	60–89 (*n* = 172)	≥90 (*n* = 132)
		Dialysis (*n* = 11)	w/o dialysis (*n* = 13)			
Age at ASCT, median years (range)	60 (32–74)	56 (38–66)	60 (43–66)	63 (45–74)	61 (33–74)	59 (32–71)
Time from Dx to ASCT, median (months)	10.4 (4.6–143.8)	11.2 (7.9–25)	9.6 (6.9–34.2)	10.9 (6–143.8)	11.1 (4.6–113.7)	8.8 (6.1–122.5)
Year of ASCT, median (range)	2011 (2007–2014)	2011 (2008–2013)	2010 (2007–2014)	2009 (2007–2014)	2010 (2007–2014)	2012 (2007–2014)
Gender (F/M)	148/222	5/6	6/7	18/24	72/100	47/85
Type of paraprotein						
IgG	225	3	4	22	106	90
IgA	63	1	1	8	32	21
IgD	1	0	0	0	0	1
kappa light chain	42	4	5	5	18	10
lambda light chain	24	3	3	4	9	5
non-secretory	4	0	0	1	2	1
unknown	11					
No of lines prior to ASCT, (%)						
1	296 (80%)	6 (54%)	10 (77%)	33 (79%)	139 (81%)	108 (82%)
>1	70 (19%)	5 (46%)	3 (23%)	8 (19%)	30 (17%)	24 (18%)
Last line of chemo						
Thalidomide based	230	6	8	27	107	82
Bortezomib based	79	4	4	8	31	32
Lenalidomide based	27	0	1	3	11	12
Thalid/Bortez based	6	1	0	0	2	3
No novel agent	24	0	0	3	18	3
Unknown *n* = 4						
Disease status at ASCT, (%)						
CR	98 (26%)	4	2	14	48	30
VGPR	88 (24%)	0	5	9	37	37
PR	113 (31%)	5	3	9	48	48
MR	18 (5%)	1	2	4	9	2
unknown	53 (14%)					

Appear on the table. These are categorised according to the degree of renal impairment (eGFR, ml/min/1.73 m^2^): <30 with or without dialysis, 30–59, 60–89, ≥90) at the time of transplant. The numbers represent the median values (range).

*eGFR* estimated glomerular filtration rate, *ASCT* autologous stem cell transplant, *Dx* diagnosis, *n* number of patients, *CR* complete response, *VGPR* very good partial response, *PR* partial response, *MR* minimal response.

The eGFR data were available for all 370 patients at the time of transplant but for only 328 patients at D + 100 and 307 patients at one-year post transplant (Supp Fig. 1). 36% (*n* = 132) had an eGFR ≥90 ml/min/1.73 m^2^, 46% (n = 172) had eGFR 60–89 ml/min/1.73 m^2^, 11% (*n* = 42) had eGFR 30–59 ml/min/1.73 m^2^ (iii) and 7% (*n* = 24) had eGFR <30 ml/min/1.73 m^2^ including 11 requiring dialysis at the time of transplant D0 (Table 1).

### Transplant characteristics and engraftment

Details of transplant characteristics are presented in Table 2. The median CD34^+^ cell dose infused was 4.4 × 10^6^ cells/kg (range 1.74–10.8) with the median number of bags being 3 (range 1–11). 83% of patients (*n* = 308) received 200 mg/m^2^ of melphalan with 17% (*n* = 62) receiving either 140 or 100 mg/m^2^.

**Table 2:** The table presents the transplant characteristics of 370 MM patients.

Patients	Total (*n* = 370)	eGFR (ml/min/1.73 m^2^)				
		<30		30–59 (*n* = 42)	60–89 (*n* = 172)	≥90 (*n* = 132)
		dialysis (*n* = 11)	w/o dialysis (*n* = 13)			
Mobilisation						
No of CD34^+^ cells (×10^6^ cells/kg)	4.4 (1.74–10.8)	3.97 (2.47–8.43)	4.2 (1.93–6.32)	4.27 (1.93–8)	4.39 (1.74–9.79)	4.49 (2.27–10.8)
No of bags (range)	3 (1–11)	7 (3–11)	6 (2–10)	3 (1–11)	4 (1–10)	3 (1–9)
Conditioning						
Melphalan 140/100 mg/m^2^ (No of pts)	62 (17%)	11	12	19	14	6
Melphalan 200 mg/m^2^ (No of pts)	308 (83%)	0	1	23	158	126
Engraftment						
Time to neutrophil engraftment (range, days)	15 (10–52)	13 (10–16)	13 (11–25)	14 (11–39)	14 (10–35)	15 (11–52)
Time to platelet engraftment (range, days)	18 (9–69)	16 (10–32)	16 (13–32)	17 (9–40)	18 (9–69)	19 (10–35)

These are subdivided into five categories, based on the renal function at the time of transplant, as follows: eGFR: <30 with or without dialysis, 30–59, 60–89, ≥90). The ‘time to neutrophil or platelet engraftment’ corresponds to the number of days post-transplant required to achieve engraftment.

*N* number of patients, *w/o* without, *No* number. Results are expressed as median values (range).

The median times to neutrophil and platelet engraftment were 15 days (range 10–52) and 18 days (range 9–69), respectively, with no patients having graft failure. There was no significant difference in engraftment times between the different renal cohorts.

### Renal outcomes post ASCT

**Fig. 1:**
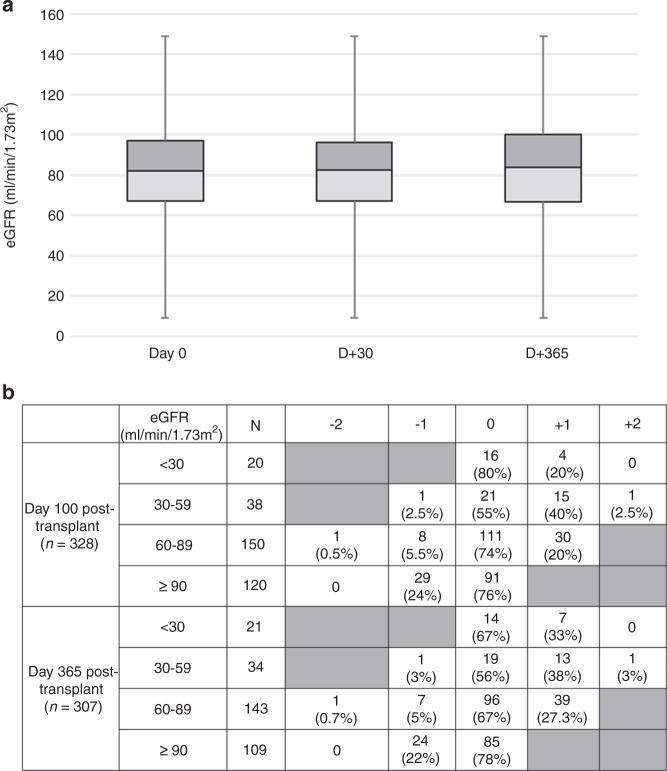
Renal response of patients with multiple myeloma following ASCT. **a** Box and Whisker plot illustrating the median eGFR at the time of transplant (Day 0) and at days 30 (D + 30) and 365 (D + 365) post-transplant. **b** Table displaying change in eGFR categories between D0 and D + 100, and D0 and D + 365 post transplant. A onestep increase in eGFR category is denoted by +1 and a two-step increase by +2; −1 and −2 represent a one and twostep decrease in eGFR category respectively. A total of 45 patients improved their renal function to an eGFR of 60–90 ml/min/1.73 m^2^ category at D + 100 and 52 patients at D + 365 after transplant. Two patients normalised their renal function after transplant. There were only two patients with a drop in eGFR to <30 ml/min/1.73m^2^ at both D + 100 and D + 365 post-transplant, suggesting an at least safe, if not beneficial, impact of autologous transplant on renal function. N number of patients.

64% of patients (*n* = 238) had an eGFR <90 ml/min/1.73 m^2^ at the time of transplant. No significant difference in renal function was noted when the median eGFR of the entire cohort was compared between the time of transplant and D + 100 or D + 365 post-transplant (Fig. 1a). However, when the individual change in eGFR between D0 and D + 100 or D + 365 was compared by paired analysis, a significant improvement in renal function at 1-year post post-transplant was observed (*p* = 0.02). Importantly no significant deterioration in renal function was seen even in patients with moderate or severe CKD and no patients became dialysis dependent post-transplant. Furthermore, no significant differences in co-morbidities were noted in different groups (Supp Table 1).

Assessment of renal response using the IMWG renal response criteria, showed that 15% (*n* = 7) of patients had a complete response, 42% (*n* = 5) had a minimal response with eGFR improvement from <15 ml/min/1.73 m^2^ to 15–30 ml/min/1.73 m^2^ and 58% of patients (*n* = 7) had a minimal response with eGFR improving from 15–30 ml/min/1.73 m^2^ to 30–60 ml/min/1.73 m^2^ (Supp Table 4).

### Effect of ASCT on dialysis dependence

Eleven patients were dialysis dependent at the time of transplant and their characteristics are shown in Table 1. 64% (*n* = 7) of these patients became dialysis free post-transplant, with four receiving a kidney transplant successfully. Three out of those four patients were still on dialysis when they received the renal transplant, but one had become dialysis independent prior to the renal transplant. All four patients had achieved a CR post ASCT. 18% (*n* = 2) of patients required less frequent dialysis sessions post ASCT, due to improvements in biochemistry and fluid balance. No significant difference in OS was noted between those patients with advanced CKD that required dialysis (*n* = 11) and those who did not (*n* = 13).

### Clinical response to ASCT and survival rates

**Fig. 2:**
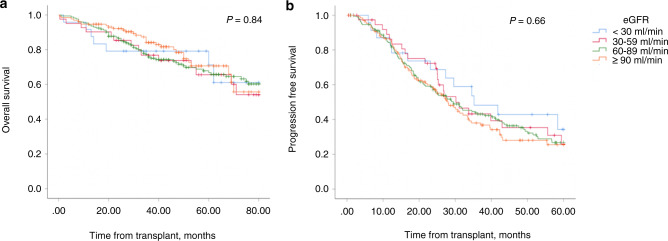
Outcomes of multiple myeloma patients following ASCT. **a** Overall survival and **b** progression free survival. Kaplan–Meier estimates showing no significant difference between the various eGFR groups (*p* = 0.84 and *p* = 0.66, respectively).

The 5-year OS post ASCT was 71%, 66%, 67% and 71% in patients with eGFR <30, 30–59, 60–89 and ≥90 ml/min/1.73 m^2^ at D0, respectively, showing no significant difference (*p* = 0.69) (Table 3). Similarly, the 5-year PFS ranged from 26 to 34% but no significant difference was noted between the different eGFR groups (*p* = 0.66) (Fig. 2). In addition, TRM at D + 100 did not vary significantly between the various eGFR groups (4.2 vs 5.1% vs 0.6 vs 1.5% for the eGFR <30, 30–59, 60–89 and ≥90 ml/min/1.73 m^2^ groups, respectively, *p* = 0.18), (Table 3).

**Table 3:** Overall survival (OS) and progression-free survival (PFS) at 5 years is illustrated, along with Transplant related mortality (TRM) at D + 100 post ASCT.

	*N*	eGFR (ml/min/1.73 m^2^)				
		<30	30–59	60–89	≥90	*p* value
OS at 5 years, %	368	71%	66%	67%	71%	0.69
PFS at 5 years, %	363	34%	26%	27%	26%	0.66
TRM at D100, %	363	4.2%	5.1%	0.6%	1.5%	0.18

No statistically significant difference was noted in OS, PFS or TRM between the different renal cohorts.

**Fig. 3:**
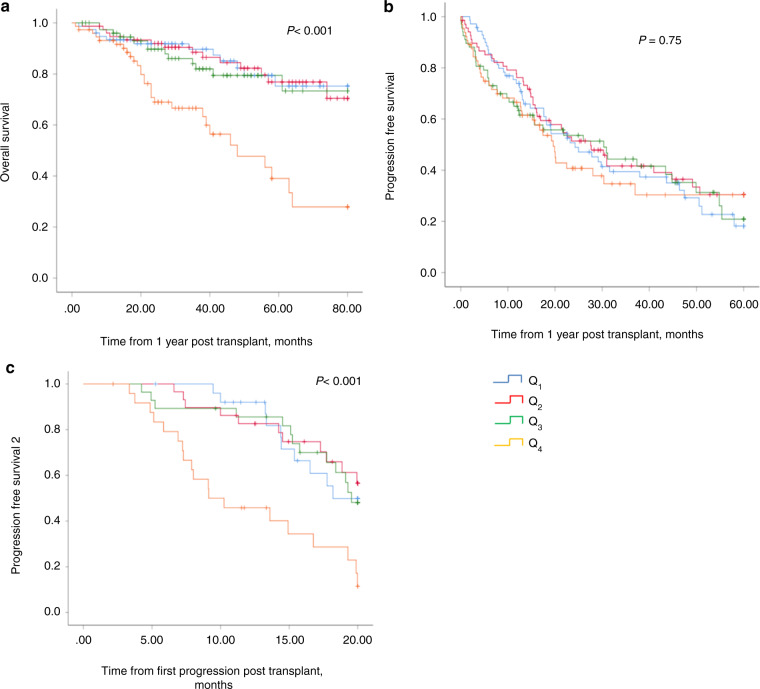
Outcomes of patients with multiple myeloma based on individual eGFR change between Day 0 and Day + 365 following ASCT. **a** Overall survival, **b** progression free survival and **c** progression free survival 2 (PFS2). A significantly worse OS was seen in *Q*_4_ (>8.79% worsening eGFR group) (*p* < 0.001) but no significant difference was noted in PFS at one year post transplant between the four quartiles (*p* = 0.75). PFS2 in a subpopulation of patients (*n* = 108) showed a significant difference between *Q*_4_ and the remaining cohorts (*Q*_1–3_) (*p* < 0.001).

In an effort to better assess the impact of renal function on transplant outcomes for each patient, the individual change in eGFR between D0 and D + 365 was calculated and grouped into quartiles (*Q*_1–4_), as described in the statistical methods. OS, PFS and PFS2 were then compared between the four quartiles. Using this analysis, it was shown that patients who had an eGFR reduction >8.79% (Q_4_ group) had a significantly worse OS compared to the other three quartiles (Fig. 3a). No significant difference was noted in PFS at 1-year post transplant between the 4 quartiles (Fig. 3b). However, a PFS2 subanalysis, done on patients where data on second relapse was available (*n* = 108), did demonstrate a statistically significant reduction in PFS2 in the cohort with >8.79% reduction in eGFR (Q_4_ group), (*p* < 0.001), (Fig. 3c). No difference in patient or transplant characteristics was noted between the 4 quartiles (Supp Table 3). Although a higher proportion of patients in Q_1_ received the lower dose of melphalan of 140 mg/m^2^, there was no correlation between the dose of melphalan and OS, PFS or PFS2 within any of the cohorts (Supp Fig. 2). Similarly, whilst there were more dialysis patients in Q1 this did not impact OS or PFS (data not shown).

## Discussion

Autologous stem cell transplantation with high dose melphalan is the standard of care for patients with MM with numerous studies showing better outcomes when used as consolidation treatment [19, 27–30]. However, the use of ASCT in MM patients with reduced eGFR has been the subject of intense debate with many centres not routinely considering such patients for transplant because of the results of early studies conducted in the pre-novel agent era showing increased morbidity and mortality rates [15, 16, 31]. There is now an increasing body of evidence suggesting that ASCT is a safe option even for MM patients with CKD [31–34]. Badros et al. [17] reported a similar median OS between patients with normal and impaired renal function (defined as Creatinine >176.8 umol/l), with no significant difference in outcomes when a lower dose of melphalan of 140 mg/m^2^ was used. Sweiss et al. [35] showed similar tolerability and efficacy of 200 mg/m^2^ melphalan in patients with moderate renal impairment (Creatinine Clearance 30–60 ml/min).

In this large study of MM patients undergoing their first ASCT following induction treatment, we compared the outcomes of patients with reduced eGFR to those with normal renal function, and also examined the impact of ASCT on long term changes in eGFR. An important strength of our study was that all transplant eligible MM patients in a defined time period undergoing ASCT were included in the analysis irrespective of their renal function at the time of transplant, in contrast to other studies that focused selectively on MM patients with reduced renal function [31–33, 36].

This study clearly demonstrates the safety of performing ASCT in MM patients with CKD including those requiring dialysis with no negative impact on neutrophil or platelet engraftment or increase in TRM observed. Furthermore, there was no difference in OS and PFS between patients with and without reduced eGFR even in those with more advanced CKD, in keeping with results from other studies [31, 37, 38].

The impact of ASCT on renal function was assessed by comparing the eGFR at the time of transplant with subsequent levels at D + 100 and D + 365 post-transplant. This is in contrast to other studies which have generally compared eGFR at the time of MM diagnosis with post-transplant levels [33]. This is important, as much of the reversibility of renal impairment in patients with MM presenting with RI occurs early during induction treatment so the beneficial effect of ASCT on renal function may have been underestimated in previous studies [10]. In order to exclude the effect of prior induction treatment on renal dysfunction, we used renal function at the time of transplant as the baseline for our analysis.

Our study showed that a substantial number of patients with reduced eGFR had an increase in eGFR by Day+100 and Day+365 after transplant. Importantly, very few patients with advanced renal disease showed worsening of renal function, and no patient commenced dialysis as a consequence of the transplant. This is in agreement with other studies showing an improvement in renal function in MM patients with reduced eGFR post ASCT [20, 33, 37, 39].

Approximately two thirds of patients who required dialysis at the time of transplant in this study became dialysis independent post-transplant, including four of them who successfully had a renal transplant [40]. Similar results were demonstrated by Bernard et al. [31] who showed 21% (7/33) patients achieving dialysis independence post ASCT and Lee et al. [32] who showed 28% (13/59) of their patients becoming dialysis independent. In a study by Mahindra et al. evaluating transplant outcomes in patients with various degrees of RI no TRM was observed at D + 100 post ASCT and 34/35 patients who were dialysis dependent at the time of transplant achieved subsequent dialysis independence. In contrast, Parikh et al. [33] showed that none of their dialysis dependent patients managed to attain independence from dialysis.

One of the striking findings of our study was that the cohort of patients with the worst eGFR deterioration at 1-year post ASCT (Q_4_) had a significantly inferior OS compared to the other cohorts. The baseline characteristics of the four groups were similar supporting the hypothesis that the observed difference in outcomes was due to changes in eGFR and not to other confounding factors. This is the first time to our knowledge, that such an impact on OS has been reported. The inferior OS was not due to early disease relapse as PFS in this cohort was not affected, however, there was a significant reduction in PFS2 suggesting that subsequent treatment after relapse may not have been as effective. Unfortunately, a multivariate analysis could not be performed to assess for other factors (such as tumour burden, co-morbidities, prior treatments, etc) that may have potentially affected survival in this group of patients due to missing data. Previous studies have suggested pre-existing CKD, raised beta-2 microglobulin (reflecting a high tumour burden), and the presence of mucositis grade 3 or 4 as independent prognostic factors for developing CKD after ASCT have been associated with increased morbidity [6]. Our data suggest that renal deterioration post ASCT is a useful biomarker to identify patients with an inferior prognosis and may be a predictor for poor response or poor tolerability to subsequent treatment.

In summary, this study confirms that ASCT is a safe and effective treatment strategy in MM patients with reduced eGFR, including those with advanced CKD and those requiring dialysis, with no increase in transplant-related mortality and equivalent progression-free and overall survival when compared to patients with normal renal function. Patients who have a significant reduction in eGFR (>8.79%) at 1-year post transplant, compared to baseline, have significantly worse overall survival and we recommend that these patients be monitored more closely.

## Supplementary information

Supplemental material

## Data Availability

The data that support the findings of this study are available from the corresponding author, RB, upon reasonable request.

## References

[1] Dimopoulos MA, Kastritis E, Rosinol L, Blade J, Ludwig H (2008). Pathogenesis and treatment of renal failure in multiple myeloma. Leukemia..

[2] Alexanian R (1990). Renal failure in multiple myeloma. Pathogenesis and prognostic implications. Arch Intern Med.

[3] Torra R (1995). Patients with multiple myeloma requiring long-term dialysis: presenting features, response to therapy, and outcome in a series of 20 cases. Br J Haematol.

[4] Blade J (1998). Renal failure in multiple myeloma: presenting features and predictors of outcome in 94 patients from a single institution. Arch Intern Med.

[5] Augustson B (2005). Earlymortality after diagnosis of multiple myeloma: analysis of patients entered ontothe United kingdom Medical Research Council trials between 1980 and 2002—Medical Research Council Adult Leukaemia Working Party. J Clin Oncol.

[6] Andronesi AG (2019). Incidence and risk factors for acute kidney injury following autologous stem cell transplantation for multiple myeloma. Cancer Med.

[7] Knudsen LM, Hjorth M, Hippe E (2000). Renal failure in multiple myeloma: reversibility and impact on the prognosis. Nordic Myeloma Study Group. Eur J Haematol.

[8] Kastritis E, Anagnostopoulos A, Roussou M, Gika D, Matsouka C, Barmparousi D (2007). Reversibility of renal failure in newly diagnosed multiple myeloma patients treated with high dose dexamethasone-containing regimens and the impact of novel agents. Haematologica..

[9] Blade J, Sonneveld P, San Miguel JF, Sutherland HJ, Hajek R, Nagler A (2008). Pegylated liposomal doxorubicin plus bortezomib in relapsed or refractory multiple myeloma: efficacy and safety in patients with renal function impairment. Clin Lymphoma Myeloma.

[10] Dimopoulos MA, Terpos E, Chanan-Khan A, Leung N, Ludwig H, Jagannath S (2010). Renal impairment in patients with multiple myeloma: a consensus statement on behalf of the International Myeloma Working Group. J Clin Oncol.

[11] Dimopoulos MA, Roussou M, Gavriatopoulou M, Psimenou E, Eleutherakis-Papaiakovou E, Migkou M (2016). Bortezomib-based triplets are associated with a high probability of dialysis independence and rapid renal recovery in newly diagnosed myeloma patients with severe renal failure or those requiring dialysis. Am J Hematol.

[12] Kumar S (2008). Improved survival in multiple myeloma and the impact of novel therapies. Blood..

[13] Gonsalves WI, Leung N, Rajkumar SV, Dispenzieri A, Lacy MQ, Hayman SR (2015). Improvement in renal function and its impact on survival in patients with newly diagnosed multiple myeloma. Blood Cancer J.

[14] Kastritis E (2009). Improved survival of patients with multiple myeloma after the introduction of novel agents and the applicability of the International Staging System (ISS): an analysis of the Greek Myeloma Study Group (GMSG). Leukemia..

[15] Carlson K (2005). Melphalan 200 mg/m^2^ with blood stem cell support as first-line myeloma therapy: impact of glomerular filtration rate on engraftment, transplantation-related toxicity and survival. Bone Marrow Transpl.

[16] Tricot G, Alberts DS, Johnson C, Roe DJ, Dorr RT, Bracy D (1996). Safety of autotransplants with high-dose melphalan in renal failure: a pharmacokinetic and toxicity study. Clin Cancer Res.

[17] Badros A, Barlogie B, Siegel E, Roberts J, Langmaid C, Zangari M (2001). Results of autologous stem cell transplant in multiple myeloma patients with renal failure. Br J Haematol.

[18] Child JA, Morgan GJ, Davies FE, Owen RG, Bell SE, Hawkins K (2003). High-dose chemotherapy with hematopoietic stem-cell rescue for multiple myeloma. N Engl J Med.

[19] Palumbo A (2013). A phase III study of ASCT vs cyclophosphamidelenalidomide-dexamethasone and lenalidomideprednisone maintenance vs lenalidomide alone in newly diagnosed myeloma patients. Blood..

[20] San Miguel JF, Lahuerta JJ, Garcia-Sanz R, Alegre A, Blade J, Martinez R (2000). Are myeloma patients with renal failure candidates for autologous stem cell transplantation?. Hematol J.

[21] Mahindra A (2017). Autologous hematopoietic cell transplantation for multiple myeloma patients with renal insufficiency: a center for international blood and marrow transplant research analysis. Bone Marrow Transplant.

[22] Kooman JP (2009). Estimation of renal function in patients with chronic kidney disease. J Magn Reson Imaging.

[23] Levey AS, Eckardt KU, Tsukamoto Y, Levin A, Coresh J, Rossert J (2005). Definition and classification of chronic kidney disease: a position statement from Kidney Disease: Improving Global Outcomes (KDIGO). Kidney Int.

[24] Ludwig H (2008). Bortezomib doxorubicin-dexamethasone (BDD) for reversal of acute light chain induced renal failure (ARF) in multiple myeloma (MM): results from a phase II study. Blood.

[25] Rajkumar SV, Harousseau JL, Durie B, Anderson KC, Dimopoulos M, Kyle R (2011). Consensus recommendations for the uniform reporting of clinical trials: report of the International Myeloma Workshop Consensus Panel 1. Blood..

[26] Suggestions on the use of statistical methodologies in studies of the European Group for Blood and Marrow Transplantation. Bone Marrow Transplant. 2013;48 Suppl 1:S1-37. 10.1038/bmt.2012.282 23462821

[27] Child J. Medical Research Council Adult Leukaemia Working Party. High-dose chemotherapy with hematopoietic stem-cell rescue for multiple myeloma. 2003. 10.1056/NEJMoa022340 12736280

[28] Attal M, Harousseau JL, Stoppa AM, Sotto JJ, Fuzibet JG, Rossi JF (1996). A prospective, randomized trial of autologous bone marrow transplantation and chemotherapy in multiple myeloma. Intergroupe Francais du Myelome. N Engl J Med.

[29] Costa LJ, Zhang MJ, Zhong X, Dispenzieri A, Lonial S, Krishnan A (2013). Trends in utilization and outcomes of autologous transplantation as early therapy for multiple myeloma. Biol Blood Marrow Transpl.

[30] Gertz MA, Dingli D (2014). How we manage autologous stem cell transplantation for patients with multiple myeloma. Blood..

[31] St Bernard R, Chodirker L, Masih-Khan E, Jiang H, Franke N, Kukreti V (2015). Efficacy, toxicity and mortality of autologous SCT in multiple myeloma patients with dialysis-dependent renal failure. Bone Marrow Transpl.

[32] Lee CK, Zangari M, Barlogie B, Fassas A, van Rhee F, Thertulien R (2004). Dialysis-dependent renal failure in patients with myeloma can be reversed by high-dose myeloablative therapy and autotransplant. Bone Marrow Transpl.

[33] Parikh GC, Amjad AI, Saliba RM, Kazmi SM, Khan ZU, Lahoti A (2009). Autologous hematopoietic stem cell transplantation may reverse renal failure in patients with multiple myeloma. Biol Blood Marrow Transpl.

[34] Tosi P, Zamagni E, Ronconi S, Benni M, Motta MR, Rizzi S (2000). Safety of autologous hematopoietic stem cell transplantation in patients with multiple myeloma and chronic renal failure. Leukemia..

[35] Sweiss K, Patel S, Culos K, Oh A, Rondelli D, Patel P (2016). Melphalan 200 mg/m2 in patients with renal impairment is associated with increased short-term toxicity but improved response and longer treatment-free survival. Bone Marrow Transpl.

[36] Tauro S, Clark FJ, Duncan N, Lipkin G, Richards N, Mahendra P (2002). Recovery of renal function after autologous stem cell transplantation in myeloma patients with end-stage renal failure. Bone Marrow Transpl.

[37] Van den Bosch I (2019). Multiple myeloma and kidney transplantation: the beginning of a new era. Clin Kidney J.

[38] Knudsen LM, Nielsen B, Gimsing P, Geisler C (2005). Autologous stem cell transplantation in multiple myeloma: outcome in patients with renal failure. Eur J Haematol.

[39] Glavey SV, Gertz MA, Dispenzieri A, Kumar S, Buadi F, Lacy M (2013). Long-term outcome of patients with multiple [corrected] myeloma-related advanced renal failure following auto-SCT. Bone Marrow Transpl.

[40] Shah S, Ibrahim M, Delaney M, Schey S, Bygrave C, Streetly M (2019). Risk of relapse of multiple myeloma following kidney transplantation. Clin Kidney J.

