# Supplementary material for: Autologous stem cell transplantation for multiple myeloma patients with chronic kidney disease: a safe and effective option
Source: Bone Marrow Transplant. 2022 Apr 12;57(6):959–65. doi: 10.1038/s41409-022-01657-y (PMC9200631; doi:10.1038/s41409-022-01657-y)
Supplement: Supplementary file 1 — Supplemental material [file 41409_2022_1657_MOESM1_ESM.docx]

**
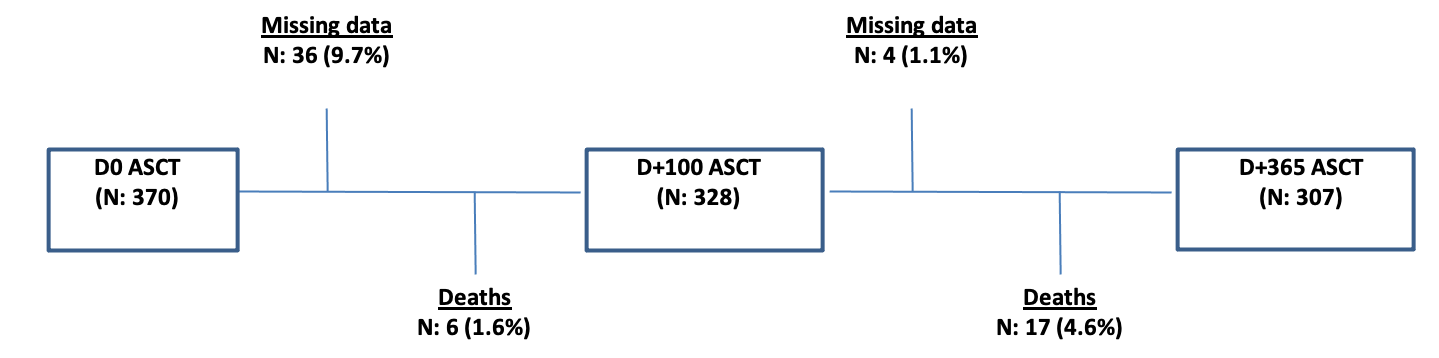
**

**Supp Figure 1:** Flow diagram indicating the number of patients with multiple myeloma who underwent ASCT between the years 2007 and 2014. The number of deaths along with the number of missing data between the time of data collection (D0 ASCT) and days 100 and 365 post ASCT is also illustrated.

Abbreviations: ASCT; autologous stem cell transplant, N; number of patients; D, day.

| **Parameters** | **eGFR (ml/min/1.73m2)** | | | | |
| --- | --- | --- | --- | --- | --- |
|  | **<30** | | **30-59 (n=42)** | **60-89 ( n=172)** | **≥90 (n=132)** |
|  | **dialysis  (n=11)** | **w/o dialysis  (n=13)** |  |  |  |
| **Diabetes** | 1 (0.9%) | 0 | 0 | 9 (5%) | 5 (4%) |
| **Hypertension** | 5 (45%) | 4 (31%) | 11 (26%) | 39 (23%) | 21 (16%) |
| **Both** | 0 | 0 | 1 (2%) | 7 4%) | 7 (5%) |
| **Ethnicity** |  | | | | |
| **White or White British** | 10 (91%) | 12 (92%) | 32 (76%) | 123 (72%) | 94 (71%) |
| **Asian or Asian British** | 0 | 0 | 0 | 3 (2%) | 6 (4%) |
| **Black, African, Caribbean or Black British** | 1 (9%) | 0 | 3 (7%) | 28 (16%) | 18 (14%) |
| **Mixed** | 0 | 1 (7%) | 0 | 4 (2%) | 1 (0.7%) |
| **Other** | 0 | 0 | 2 (5%) | 2 (1%) | 5 (4%) |
| **Unknown** |  |  | 5 (12%) | 12 (7%) | 7 (5%) |
| **Amyloid** | 1 (9%) | 0 | 3 (7%) | 3 (2%) | 2 (1.5%) |
| **Extramedullary disease** | 0 | 0 | 0 | 2 (1%) | 1 (0.7%) |
| **Renal Biopsy** | 6 (54%) | 6 (50%) | 4 (9%) | 1 (0.5%) | 0 |

**Supp Table 1:** The table demonstrates variable parameters/co-morbidities, as these are distributed among the various eGFR groups.

**
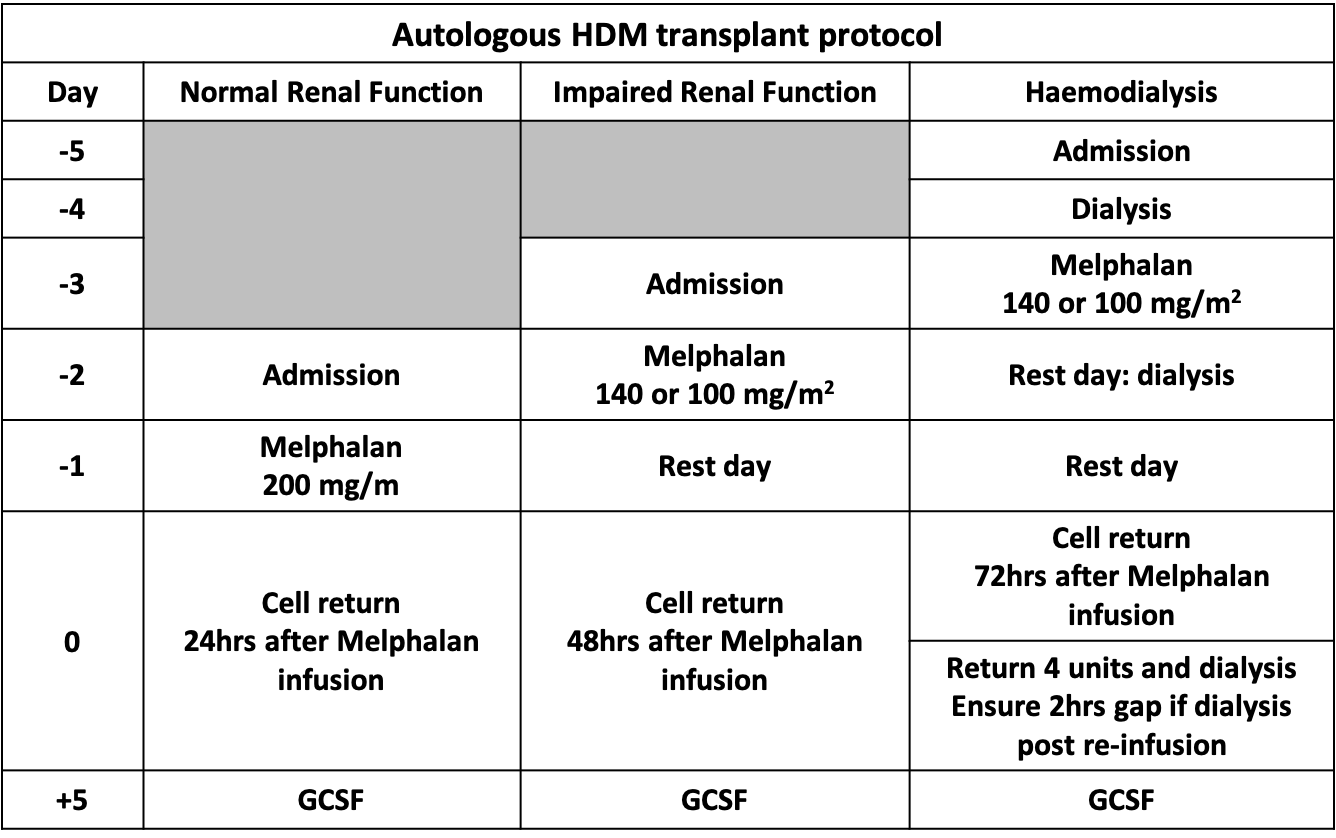
**

**Supp Table 2:** The table above illustrates the different autologous high dose melphalan (HDM) stem cell transplant protocols applied in patients with: a) normal renal function, b) impaired renal function and c) patients on haemodialysis. It is evident that patients on dialysis require a 72-hour (hr) gap after the Melphalan infusion and prior to stem cell infusion, whereas patients with renal impairment require a 48hr gap. The Melphalan dose can be further reduced to 100mg/m^2^, depending on the presence of co-existent comorbidities. For patients on haemodialysis, a repeat dialysis session is scheduled after the return of the fourth stem cell unit, with a 2hr gap between the stem cell infusion and the dialysis.


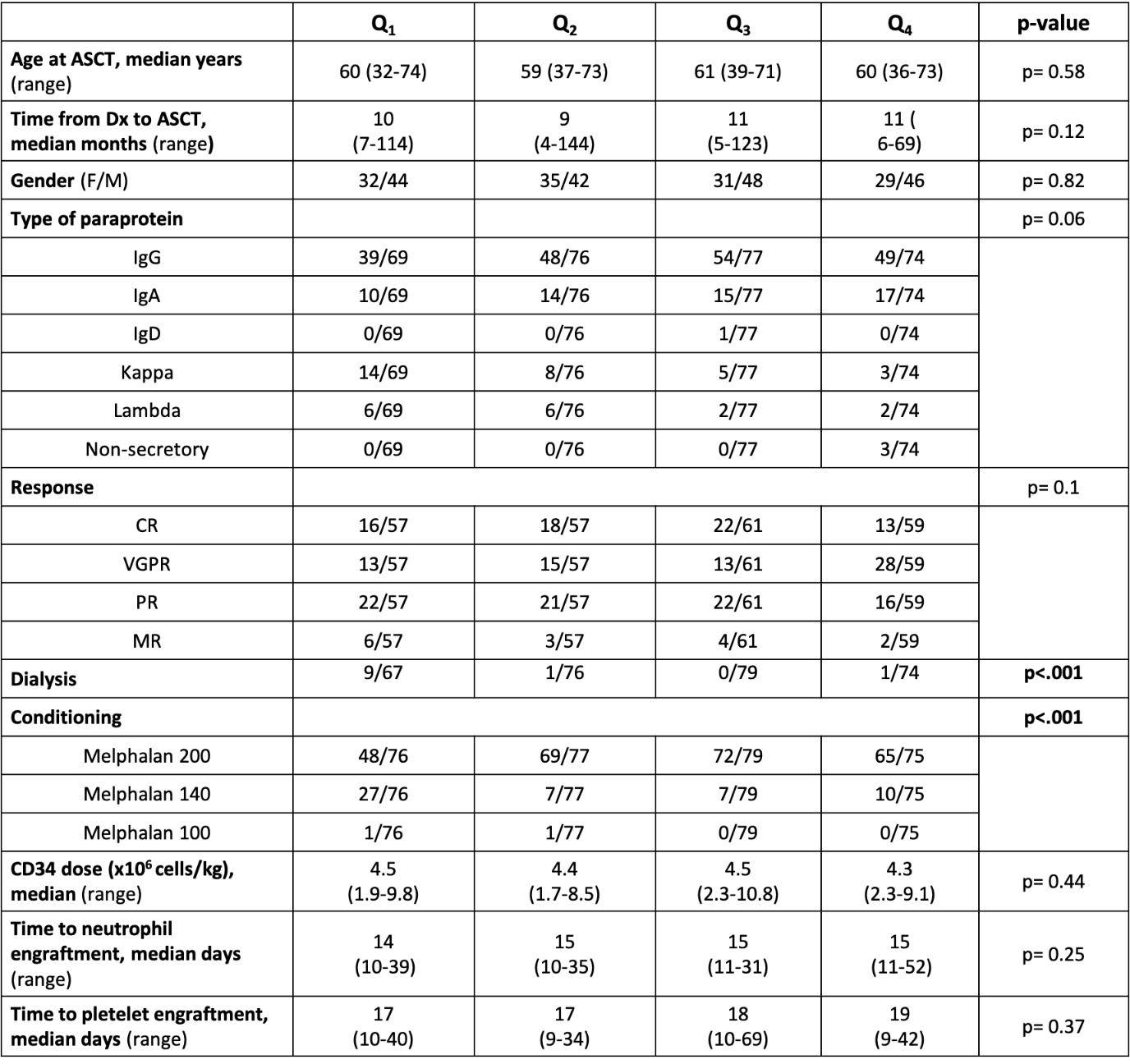


**Supp Table 3:** The table demonstrates the distribution of patients’ characteristics among the various quartiles (Q_1-4_). A significant difference was noted in the use of dialysis and the melphalan dose among the various groups, supporting the notion that the group with the highest eGFR improvement (Q_1_) is the group with the worst renal function (including the dialysis patients), that naturally received reduced melphalan doses.

**A]**


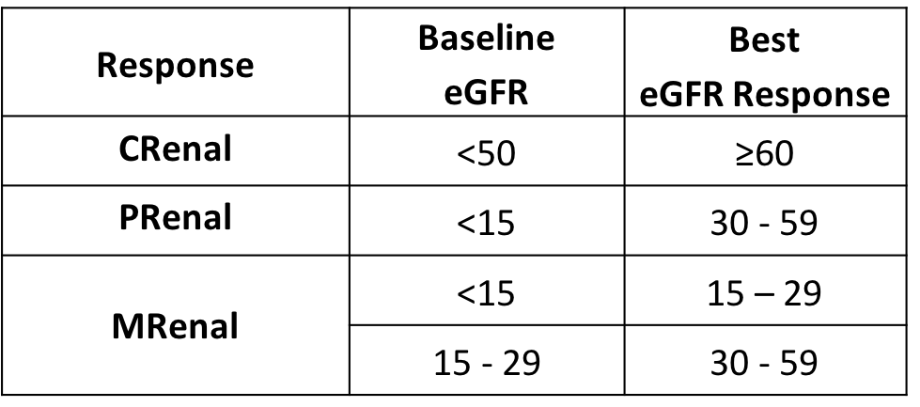


**B]**


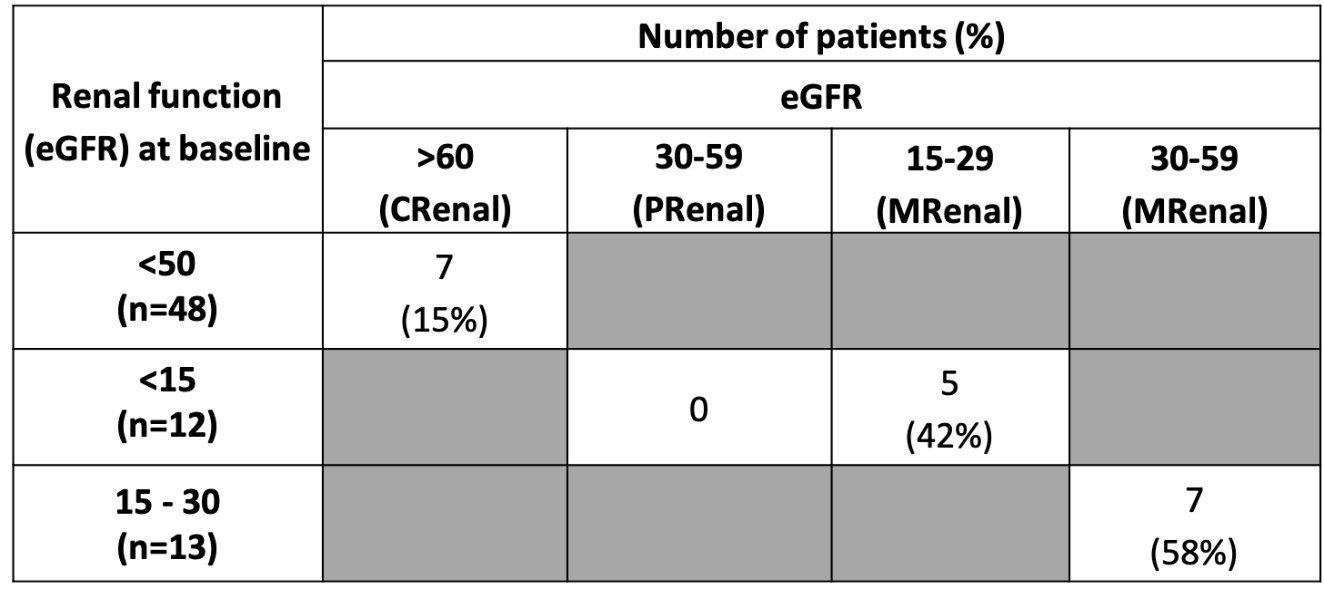


**Supp Table 4: A]** The IMWG Renal Response criteria are illustrated. Complete Renal response (CRenal) is defined as an improvement of eGFR to >60, Partial Renal response (PRenal) as an improvement of eGFR from <15 to 30-59 ml/min/1.73m^2^ and Minimal Renal response (MRenal) either as an improved from <15 to 15-30 ml/min/1.73m^2^ (MRenal i) or from 15-29 to 30-59 ml/min/1.73m^2^ (MRenal ii). **B]** The table demonstrates the IMWG-defined Renal Response after ASCT. 15% of patients with eGFR <50 achieved CRenal. None of the patients with an eGFR<15 achieved PRenal, whereas 42% of patients achieved MRenal. Of the patients with an eGFR of 15-30 ml/min/1.73m^2^, 58% achieved MRenal.


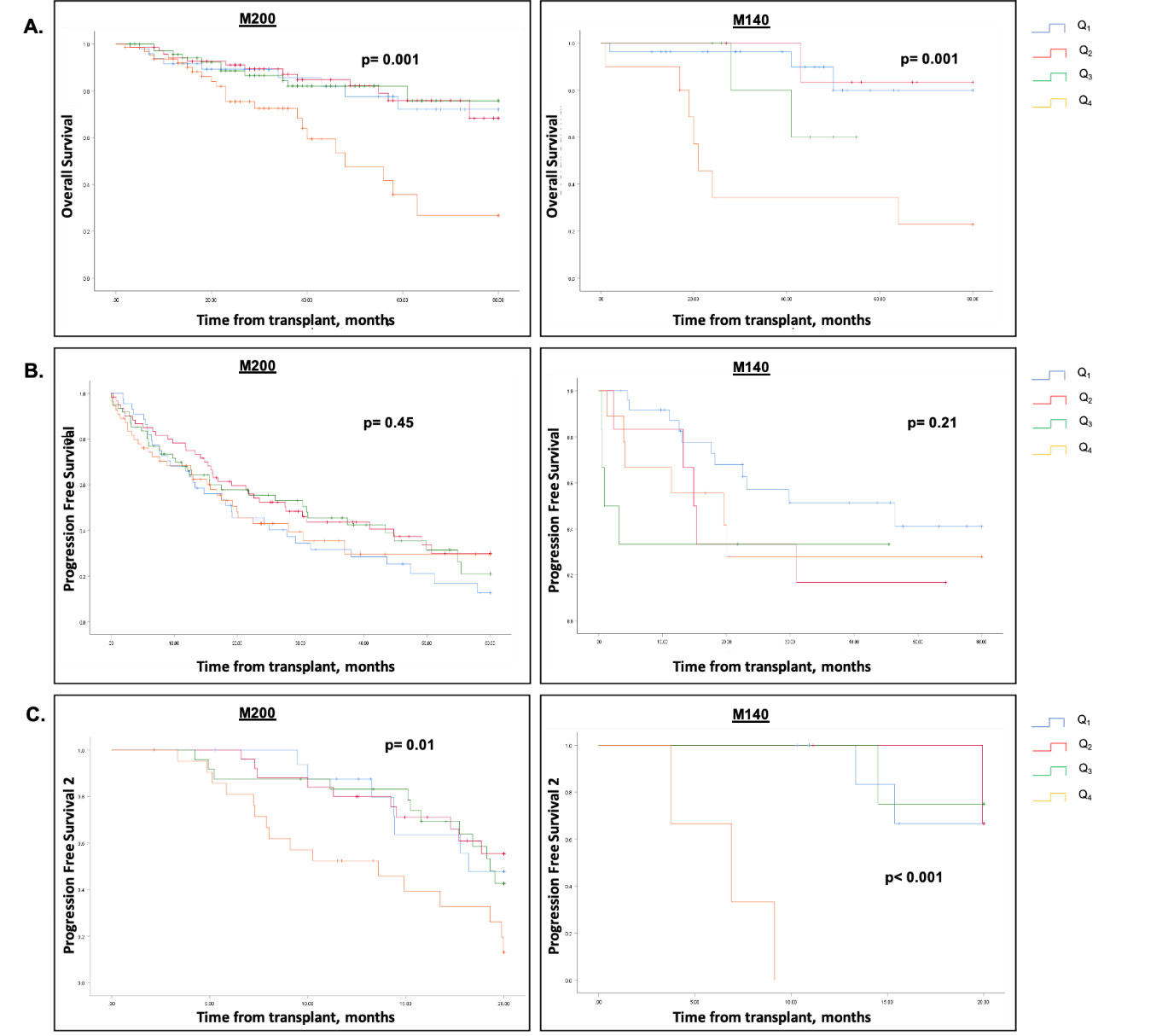


**Supp Figure 2:** The figure illustrates the Overall Survival **(A)**, Progression Free Survival **(B)** and Progression Free Survival 2 **(C)**, among patients who received a normal melphalan dose of 200 mg/m^2^ (M200) and those who received a lower dose of 140 mg/m^2^ (M140). As demonstrated, there is no difference in either OS, PFS or PFS2 between the two groups, suggesting that the impact of a declining renal function (Q_4_) on survival is not related to the melphalan dose.

Abbreviations: Q_1_; >15.4% eGFR improvement, Q_2_; 3.91 to 15.4% eGFR improvement, Q_3_; 3.91% eGFR improvement to 8.78% eGFR worsening, Q_4_; >8.79% eGFR worsening.
